# Identification of Driver Genes Regulating the T-Cell–Infiltrating Levels in Hepatocellular Carcinoma

**DOI:** 10.3389/fgene.2020.560546

**Published:** 2020-12-14

**Authors:** Yi Cai, Ying Tian, Jianchu Wang, Wang Wei, Qianli Tang, Libai Lu, Zongjiang Luo, Wenchuan Li, Yuan Lu, Jian Pu, Zhengxia Yang

**Affiliations:** ^1^Department of Oncology, Hospital of Chengdu University of Traditional Chinese Medicine, Chengdu, China; ^2^Department of Urology Surgery, Hospital of Chengdu University of Traditional Chinese Medicine, Chengdu, China; ^3^Department of Hepatobiliary Surgery, Affiliated Hospital of Youjiang Medical University for Nationalities, Baise, China; ^4^Department of Gastroenterology, Huai’an Second People’s Hospital, The Affiliated Huai’an Hospital of Xuzhou Medical University, Huai’an, China

**Keywords:** T-cell infiltration, cancer immunotherapy, immune escape, driver gene, oncogenic pathway

## Abstract

The driver genes regulating T-cell infiltration are important for understanding immune-escape mechanisms and developing more effective immunotherapy. However, researches in this field have rarely been reported in hepatocellular carcinoma (HCC). In the present study, we identified cancer driver genes triggered by copy number alterations such as *CDKN2B*, *MYC*, *TSC1*, *TP53*, and *GSK3B*. The T-cell infiltration levels were significantly decreased in both HCC and recurrent HCC tissues compared with the adjacent normal liver tissues. Remarkably, we identified that copy number losses of *MAX* and *TP53* were candidate driver genes that significantly suppress T-cell infiltration in HCC. Accordingly, their downstream oncogenic pathway, cell cycle, was significantly activated in the low T-cell infiltration HCC. Moreover, the chemokine-related target genes by TP53, which played key roles in T-cell recruitment, were also downregulated in HCC with TP53/MAX deletions, suggesting that copy number losses in *MAX* and *TP53* might result in T-cell depletion in HCC via downregulating chemokines. Clinically, the T-cell infiltration levels and chemokines activity could accurately predict the response of sorafenib, and the prognostic outcomes in HCC. In conclusion, the systematic analysis not only facilitates identification of driver genes and signaling pathways involved in T-cell infiltration and immune escape, but also gains more insights into the functional roles of T cells in HCC.

## Introduction

Hepatocellular carcinoma (HCC) is a highly aggressive cancer with an increasing incidence, accounting for the majority of all primary liver cancer cases (Kung-Chun Chiu et al., 2020). Prior hepatitis B and/or hepatitis C infection is often considered a major risk factor of HCC, along with alcohol consumption, tobacco use, and obesity (Grandhi et al., 2016). Clinically, limited early symptoms in HCC patients could lead to delayed diagnoses, which greatly undermines the survival outcomes of HCC patients (Sun et al., 2020). For patients with advanced stage HCC, whose conditions are usually not suitable for surgical resection, immunotherapies are considered as an effective and promising strategy (Caraballo Galva et al., 2020).

The association between a dysregulated immune system and the development of HCC has been demonstrated in many studies, and changes in the abundance or function of tumor-related immune cells, such as self-reactive cytotoxic T cells, CD4^+^ T cells, regulatory T cells, and natural killer (NK) cells, are observed in HCC (Ding et al., 2018). T lymphocytes are among the most critical players in the inhibition of tumor cells (Thompson et al., 2010). It has been reported that cytotoxic T lymphocytes and CD4^+^ T cells could affect antigen recognition and DNA-based immunization (Grimm et al., 2000). To our knowledge, tumor-infiltrating lymphocytes are an essential component of tumor microenvironment (TME), and a previous study has assessed the clinical significance of tumor-infiltrating NK cells in HCC, successfully relating NK cell abundances to several immune checkpoint proteins (Wu et al., 2020).

As higher T lymphocyte–infiltrating rates are considered to be associated with favorable prognoses in many cancers (Chaoul et al., 2020), it is critical to explore what potentially drives T-lymphocyte infiltration in HCC patients. Notably, genomic aberrations, such as copy number alterations (CNAs) and neoantigen load, are also observed to be associated with immune infiltration in other cancers (Safonov et al., 2017). As new computational methods have enabled the identification and interpretation of cancer driver genes at multi-omics, it is now possible to explore mechanism behind CNA-related cancer driver genes and the T-lymphocyte infiltration and to examine the association between different T lymphocytes infiltrating levels and response to antitumoral drugs and their impacts on HCC prognosis. Therefore, in order to identify the CNA-triggered driver genes and unveil their underlying molecular mechanisms involved in T-cell infiltration, we conducted a systematic data analysis and anticipated to identify the driver genes associated with T-cell infiltration, and link them to drug treatment response and prognostic outcomes.

## Materials and Methods

### Data Acquisition

The discovery datasets of gene expression and CNAs in The Cancer Genome Atlas (TCGA) cohort were collected from UCSC Xena database (Goldman et al., 2019), which curated preprocessed TCGA data including genomics, transcriptome, and proteome. Moreover, we also downloaded normalized gene expression data of GSE109211 (Pinyol et al., 2019), GSE56545 (Xue et al., 2015), and GSE14520 (Roessler et al., 2010) from Gene Expression Omnibus (GEO) database to evaluate the associations of T-cell infiltration levels with recurrence, response of sorafenib treatments, and prognostic outcomes. The normalized gene expression data from the Clinical Proteomic Tumor Analysis Consortium (CPTAC) HCC cohorts were collected from previous study (Gao et al., 2019). We also collected 242 well-known driver genes including 127 oncogenes and 115 tumor suppressors from previous study (Mayakonda et al., 2018).

### The Correlation Analysis of CNAs and Gene Expression Levels

The segmented CNAs were used as the input of GISTIC v2.0 (Mermel et al., 2011) (Genomic Identification of Significant Targets in Cancer). The recurrent CNAs identified by GISTIC were used for the correlation analysis. For each gene, Pearson correlation analysis was conducted on the log2 copy number ratio and log2-FPKM (fragment per kilobase of exon model per million reads mapped)–based gene expression. Pearson correlation coefficient of 0.4 was used as cutoff threshold to determine whether the gene expression was triggered by the CNAs. The resulting driver genes should also be curated in the 242 well-known driver genes.

### The Infiltration Levels of T Cells and Cell Cycle or Chemokine Activity

The T-cell–specific marker genes, marker genes of the seven-step cancer–immunity cycle, and gene expression data of HCC cell lines were collected from previous studies (Zheng et al., 2017; Xu et al., 2018; Qiu et al., 2019). Subsequently, the T-cell–specific marker genes were refined by excluding those with expression levels higher than 1 FPKM in HCC cell lines. We conducted single-sample gene set enrichment analysis (ssGSEA) to estimate the T-cell infiltration, cell cycle, and chemokine activity by their signature genes. The ssGSEA is a rank-based method to measure the relative abundance for a gene list of interest and has been widely used to estimate immune-cell infiltration and validated in a series of *in vitro* and *in silico* tests by previous studies (Senbabaoglu et al., 2016; Xue et al., 2019; Yu et al., 2019; Zuo et al., 2020). The ssGSEA was implemented in R GSVA v1.36.2 package (Hanzelmann et al., 2013) with *gsva* function.

### The Anticancer Immune Response of Cancer–Immunity Cycle

First, we collected signature genes from previous study (Xu et al., 2018), which defined the cancer–immunity cycle as seven steps of anticancer response. Second, we selected T cell–related gene signatures (Supplementary Table S2) and estimated the anticancer activity of the seven steps by ssGSEA.

### Gene Set Enrichment Analysis

The GSEA was conducted against Reactome pathways (Jassal et al., 2020) on the preranked gene set based on the *t* statistics, which were calculated by comparing the samples of high T-cell infiltration with those of low T-cell infiltration. The GSEA was implemented in R *fgsea* v1.14.0 package with *fgsea* function.

### Principal Component Analysis and Support Vector Machine Model

The principal component analysis (PCA) and support vector machine (SVM) were conducted on the infiltrating levels of the five T cells. The PCA was implemented and visualized in R *FactoMineR* and *factoextra* packages. The R *e1071* and *ROCR* package were employed to build the SVM model (Sing et al., 2005), visualize the receiver operating characteristic (ROC) curves, and calculate the area under the curve (AUC) values.

### Survival Analysis

The infiltrating levels of the five T cells and chemokine activity were used as the predictors of the overall survival (OS). The chemokine activity was estimated by the ssGSEA. The Cox proportional hazards regression model was employed to perform univariate and multivariate analyses for OS. A multivariable Cox model was built based on the infiltrating levels of T cells and chemokine activity, which were selected by stepwise method. The Cox model was trained in TCGA cohort and used to predict the risk scores for the samples from GSE14520 and CPTAC HCC cohorts using the corresponding T-cell–infiltrating levels and chemokine activities. The median value of the risk scores was used as cutoff thresholds to plot the KM curves, and the statistical significance was evaluated by the log–rank test.

### Statistical Analyses

All the statistical analyses were performed in R (version 4.0.0). The Wilcoxon rank–sum test or *t*-test was employed to compare the means of the two samples. Multiple sample comparison was tested by analysis of variance (ANOVA). The symbols of ^∗^, ^∗∗^, ^∗∗∗^, ^****^ represent the statistical significance at 0.05, 0.01, 0.001, and 0.0001, respectively.

## Results

### Identification of Deregulated Genes Triggered by CNAs

**FIGURE 1:**
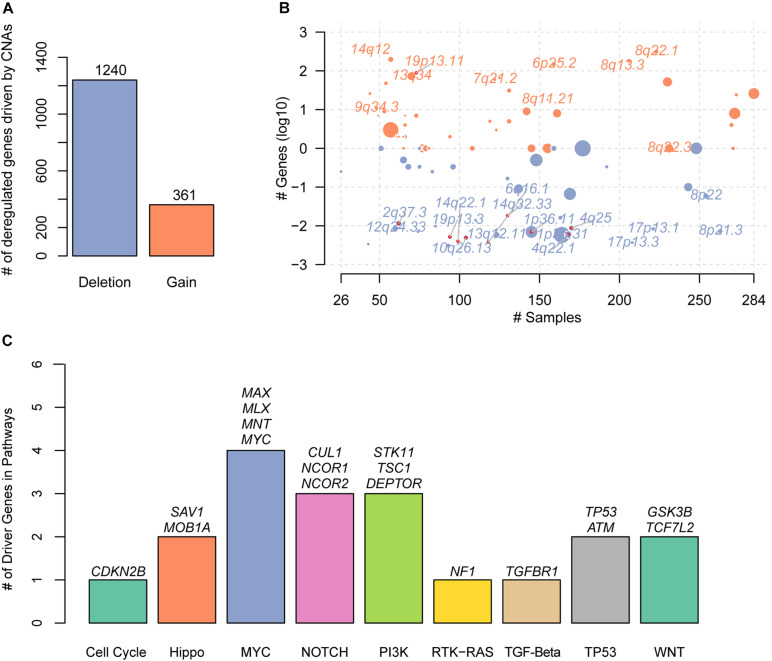
The copy number alterations (CNAs)–related driver genes in HCC. **(A)** The deregulated genes driven by the CNAs. The orange and blue bars represent the gains and deletions, respectively. **(B)** The frequent CNA cytoband of the driver genes. Size of each bubble is according to –log10 transformed *q*-values. The *x* and *y*-axes represent the number of samples with CNAs and the *q*-value of the cytoband by GISTIC. **(C)** The known driver genes also identified by the CNA-gene expression-based correlation analysis. Each bar represents an oncogenic pathway.

With the gene expression data and CNAs from TCGA HCC cohort, we systematically identified the deregulated genes triggered by conducting correlation analysis of the copy number ratio and gene expression levels for each gene. Given a threshold of Pearson correlation coefficient at 0.4, we identified 361 gains and 1,240 deletions in the recurrently altered CNA regions (Figure 1A and Supplementary Table S1), respectively. With these genes significantly regulated by the CNAs, we mapped them to the genomic regions and found that gains were frequently located within 14q12, 19p13.11, 13q34, 9q34.3, 7q21.2, 6p25.2, 8q11.21, 8q13.3, 8q22.1, and 8q22.3 (Figure 1B). The deletions were enriched in 1p36.31, 1p36.11, 4q22.1, 12q24.33, 13q12.11, 8p22, 2q37.3, 4q25, 10q26.13, 8p21.3, 19p13.3, 14q32.33, 17p13.1, 14q22.1, 17p13.3, and 6q16.1 (Figure 1B). Specifically, we found 19 known driver genes involved in 9 pathways, of which MYC had the greatest number of driver genes, including *MAX*, *MLX*, *MNT*, and *MYC* (Figure 1C). In addition, NOTCH and PI3K also had relatively more driver genes than others. These results indicated that the correlation analysis of CNAs and gene expression could efficiently identify the driver genes triggered by CNAs.

### Discovery of Driver Genes Associated With T-Cell Infiltration

**FIGURE 2:**
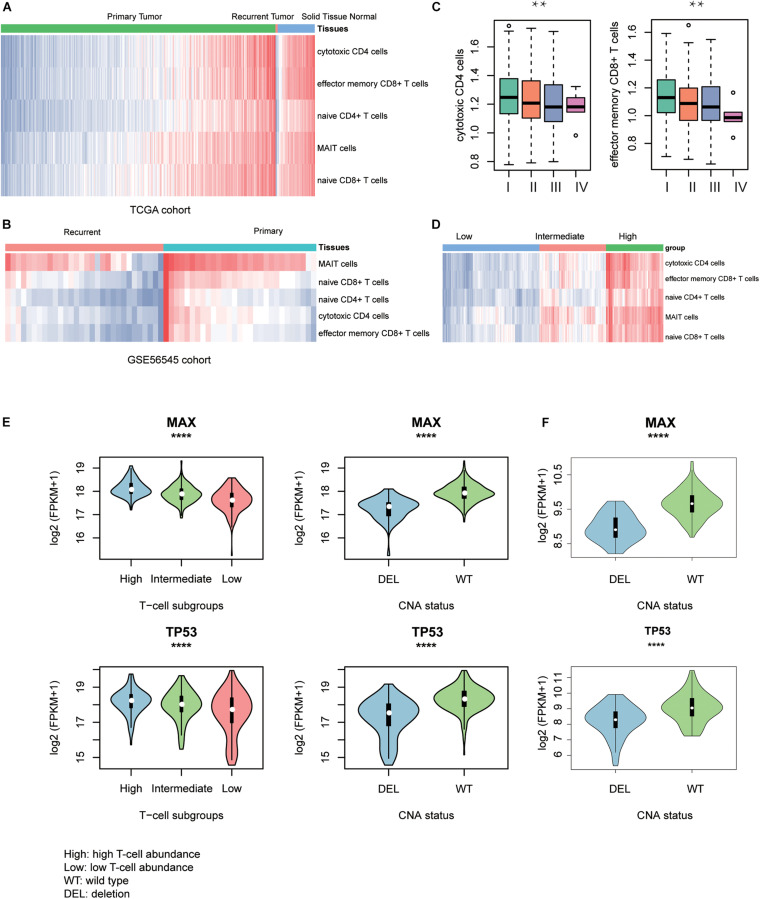
T-cell–infiltrating levels and driver genes potentially regulating T-cell infiltration. The differences of T-cell–infiltrating levels between adjacent normal liver tissues, HCC, and recurrent HCC tissues were visualized in TCGA **(A)** and GSE56545 **(B)** cohorts. **(C)** The differential infiltrating levels of cytotoxic CD4 cells and effector memory CD8^+^ T cells across TNM stages. **(D)** The unsupervised clustering of the HCC samples from TCGA based on the T-cell–infiltrating levels. The three clusters represent low (blue), intermediate (red), and high T-cell infiltration (green). The expression patterns of two driver genes, TP53 and MAX, potentially regulating the T-cell infiltration into the HCC tissues in TCGA **(E)** and CPTAC **(F)** cohorts. The symbols of *, **, ***, and **** represent the statistical significance at 0.05, 0.01, 0.001, and 0.0001, respectively.

To discover the driver genes associated with T-cell infiltration, we first estimated the infiltrating levels of five representative T cells including cytotoxic CD4 cells, effector memory CD8^+^ T cells, naive CD4^+^ T cells, mucosal-associated invariant T (MAIT) cells, and naive CD8^+^ T cells for the TCGA and GSE56545 samples by ssGSEA. Specifically, the normal tissues had significantly higher T-cell–infiltrating levels than the both primary and recurrent tumor tissues (Figure 2A, Wilcoxon rank–sum test, *P* < 0.05). Moreover, we also found that the T-cell–infiltrating levels were significantly lower in recurrent tissues compared with the primary tissues (Figure 2B, *P* < 0.05). In addition, we observed that the infiltrating levels of cytotoxic CD4^+^ and effector memory CD8^+^ cells were higher in stage I and were decreased with the degree of the disease stage (Figure 2C). Exceptionally, naive CD4/CD8 and MAIT cells were observed to be highly infiltrated into stage IV tumors, but these cells did not have anticancer effects (Supplementary Figure 1A). These results indicated that the T-cell–infiltrating levels were significantly lower in primary HCC and reduced in recurrent and advanced-stage HCC as compared with the primary tumors.

With the infiltrating levels, we classified the TCGA HCC samples into three subgroups, with high, intermediate, and low T-cell–infiltrating levels by hierarchical clustering analysis (Figure 2D), respectively. The comparative analysis of the 19 driver genes in the samples with distinct levels of T-cell infiltration or copy numbers of driver genes revealed that only two genes, *TP53* and *MAX*, were significantly downregulated in samples with either low T-cell infiltration or deletion of the corresponding genes (Figure 2E, ANOVA and textitt-test, *P* < 0.0001). Consistently, the two genes were also observed to be downregulated in CPTAC HCC samples with CNV loss of *TP53* or MAX (Figure 2F, *P* < 0.0001). These results indicated that the TP53 and MAX might be the candidate driver genes that regulate the T-cell infiltration.

### Signaling Pathways Regulating the Infiltrating Levels of T Cells in HCC

**FIGURE 3:**
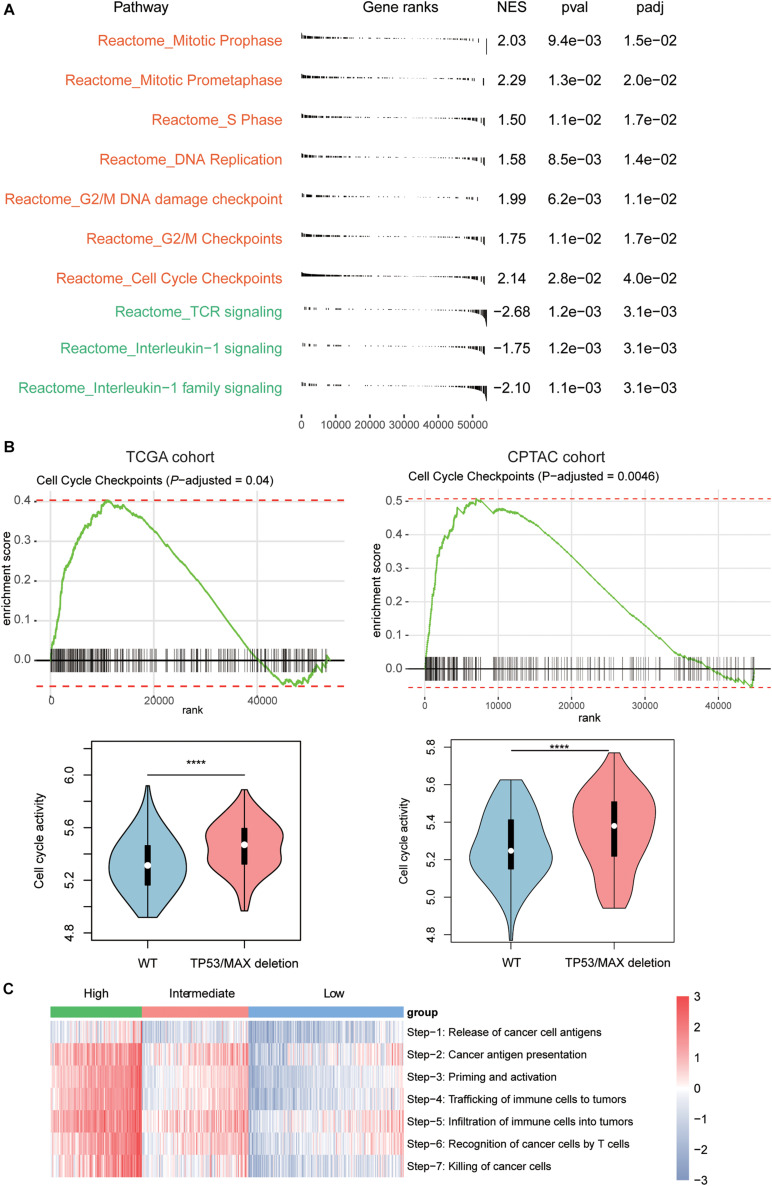
Signaling pathways involved in T-cell infiltration. **(A)** The signaling pathways enriched by the upregulated genes in low T-cell infiltration (red) and high T-cell infiltration (green) subgroups. The *P*-value and adjusted *P*-value were used to evaluate the enrichment of those genes in the pathways. **(B)** The enrichment degree of the cell cycle checkpoint and its activities between HCC samples with and without TP53/MAX deletions in TCGA (left panel) and CPTAC (right panel) cohorts. **(C)** The estimated enrichment scores of the seven-step cancer–immunity cycle in HCC samples from the three subgroups. The symbols of *, **, ***, and **** represent the statistical significance at 0.05, 0.01, 0.001, and 0.0001, respectively.

To further investigate the signaling pathways that potentially regulate the T cells infiltrating into the HCC tissues, we compared the gene expression profiles of low T-cell infiltration subgroup with those of the high T-cell infiltration subgroup. The GSEA revealed that cell cycle progression–related pathways including mitotic prophase, mitotic prometaphase, S phase, DNA replication, G2/M DNA damage checkpoint, G2/M Checkpoints, and cell cycle checkpoints were significantly upregulated in low T-cell infiltration subgroup, whereas the activities of TCR signaling, interleukin-1 signaling, and interleukin-1 family signaling were observed higher in high T-cell infiltration subgroup (Figure 3A, Benjamini and Hochberg adjusted *P* < 0.05). Moreover, as TP53 and MAX were involved in regulating the cell cycle checkpoint, the activity of cell cycle pathway was significantly enhanced in both TCGA and CPTAC HCC samples with TP53 or MAX deletions (Figure 3B, *P* < 0.0001). Furthermore, we also conducted ssGSEA to estimate the T-cell–mediated anticancer activity of the seven steps based on the signature genes by previous study (Xu et al., 2018) and found that high T-cell infiltration subgroup had higher activities of all the seven steps than the low T-cell infiltration subgroup (Figure 3C), indicating that the reduced anticancer activity mediated by T-cell infiltration in HCC might be caused by a systematic dysfunction of the entire process.

**FIGURE 4:**
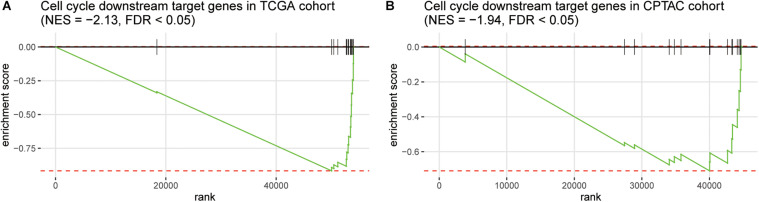
The enrichment degree of the chemokine-related target genes in the downregulated genes of HCC samples with TP53/MAX deletion. The gene set enrichment analysis (GSEA) was conducted in both TCGA **(A)** and CPTAC **(B)** cohorts. The normalized enrichment score (NES) and FDR were shown on the top of the panels.

Furthermore, as the immune-cell recruitment might be regulated by the chemokines secreted by the tumor cells in TME (Chow and Luster, 2014), we then investigated whether CNV loss of *TP53* and *MAX* could regulate the expression of the cytokines. We then collected 14 genes encoding chemokines including *CCR7*, *CCL3*, *CCL4*, *CCL19*, *CCL21*, *CXCL10*, *CXCL11*, *CCL7*, *CXCL1*, *CXCR4*, *CCL1*, *CCL17*, *CCR4*, and *CCL28*, which were also transcriptionally regulated by the transcription factors involved in cell cycle such as *TP53* and *MAX*, and found that these genes were highly enriched in the downregulated genes in HCC with *TP53/MAX* deletions (Figures 4A,B; false discovery rate (FDR) < 0.05), indicating that the CNV loss in *TP53/MAX* might downregulate their target genes, thereby weakening the immune-cell recruitment.

### T-Cell–Infiltrating Levels and Chemokines Are Associated With Treatment Response in HCC

**FIGURE 5:**
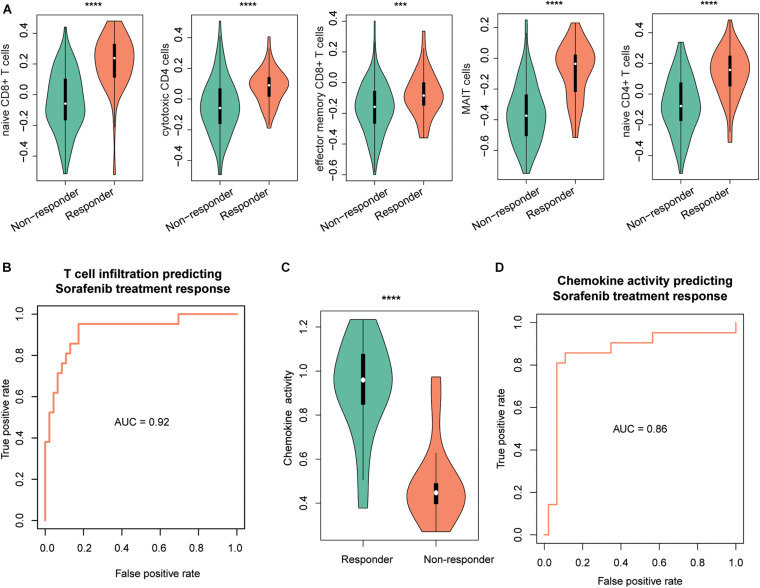
The performance of T-cell–infiltrating levels in HCC treatment response prediction. The distribution of the infiltrating levels of five T cells **(A)** and chemokine activity **(C)** in HCC responders vs. nonresponders of sorafenib. The ROC curves of the SVM models for the prediction of the responses of sorafenib treatment using T-cell infiltration **(B)** and chemokine activity **(D)**. The symbols of *, **, ***, and **** represent the statistical significance at 0.05, 0.01, 0.001, and 0.0001, respectively.

As the immune cells infiltrating into tumor tissues could enhance the chemotherapy response (Rusakiewicz et al., 2013; Hugo et al., 2015; Cai et al., 2019), we then investigated whether the two driver genes, T-cell–infiltrating levels and chemokines, were also associated with treatment response in HCC. We collected another gene expression dataset with sorafenib treatment response (GEO accession: GSE109211). As expected, TP53 and MAX were expressed higher in responders than nonresponders (Supplementary Figure 1B, *P* < 0.0001). Similarly, we estimated the infiltrating levels of five T cells and chemokine activities for the HCC samples by ssGSEA and corresponding signature genes and built two SVM models using the T-cell–infiltrating levels and chemokine activity to predict the response of sorafenib treatment, respectively. The infiltrating levels of the five T cells were higher in the responders than nonresponders (Figure 5A). The ROC curves revealed that the T-cell infiltration could accurately predict the response of both sorafenib treatment with AUC values of 0.92 (Figure 5B), respectively. Moreover, we also observed chemokine activity was higher in HCC responders than nonresponders (Figure 5C, *t*-test, *P* < 0.0001). The ROC analysis revealed that chemokine activity could also predict the response of sorafenib treatment in HCC at a high performance (Figure 5D, AUC = 0.86), indicating that chemokine activity might be associated with sorafenib sensitivity in HCC. These results indicated that the T-cell infiltration and chemokine activity in HCC could predict the response of sorafenib treatment in HCC.

### The Prognostic Significance of T-Cell–Infiltrating Levels and Cell Cycle Activity in HCC

**FIGURE 6:**
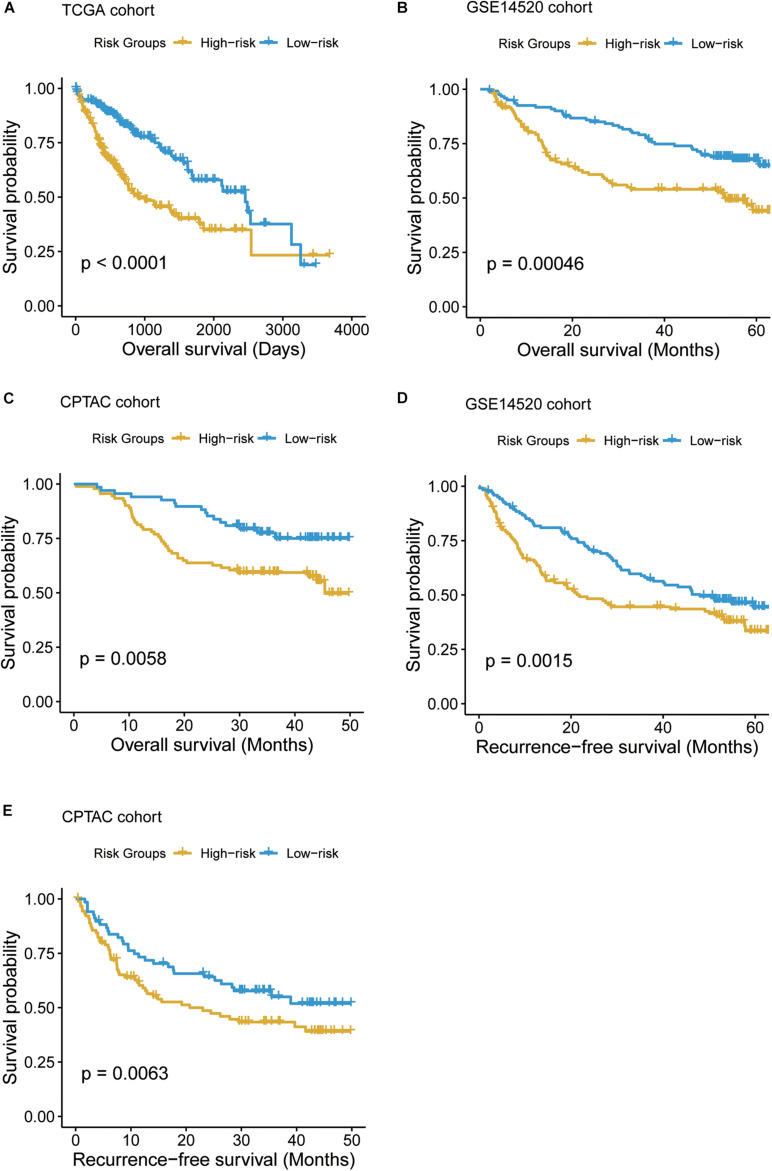
The Kaplan–Meier (KM) curves of the HCC samples stratified by the T-cell–infiltrating levels. **(A)** The overall survival probabilities of the high- and low-risk groups in TCGA cohort. The multivariable Cox model was built based on the T-cell–infiltrating levels of TCGA samples to stratify those samples by the median of the risk scores. **(B, C)** The difference of overall survival probabilities between two risk groups in GSE14520 and CPTAC cohorts. The differences of the recurrence-free survival between the two risk groups in GSE14520 **(D)** and CPTAC **(E)** cohorts.

To systematically evaluate the prognostic value of the T-cell–infiltrating levels, chemokine activity, and the two driver genes, TP53 and MAX, in HCC, we tested their association with OS in TCGA, GSE14520, and CPTAC cohorts (Gao et al., 2019). First, the cytotoxic CD4 cells, effector memory CD8^+^ T cells, MAIT cells, and chemokine activity were selected as the best combination for risk prediction by the stepwise method, and we built the multivariable Cox model based on the T-cell–infiltrating levels and chemokine activity in TCGA cohort (Table 1). The high-risk group had significantly shorter OS than the low-risk group (Figure 6A, *P* < 0.0001). Moreover, the risk scores of samples from the two testing cohorts, GSE14520 and CPTAC, were predicted based on the trained model and corresponding T-cell–infiltrating levels and cell cycle activity. Consistently, the OS in high-risk group was still worse than the low-risk group in both of the cohorts (Figures 6B,C; *P* < 0.05). With this sample stratification, the differences of recurrence-free survival (RFS) between the high- and low-risk groups were also tested. The RFS was also observed shorter in high-risk group than the low-risk group of the two testing cohorts (Figures 6D,E; *P* < 0.05). These results indicated that the T-cell–infiltrating levels and cell cycle activity could serve as potential prognostic biomarkers in HCC.

**TABLE 1:** The hazard ratio and statistical significance of the three T cells and cell cycle activity selected by stepwise method in both univariate and multivariate analyses.

Factors	Univariate analysis (95% CI)	*P*-value	Multivariate analysis (95% CI)	*P*-value
Cytotoxic CD4 cells	0.2579 (0.0973–0.6831)	0.0064	0.0138 (0.0004–0.3914)	0.012
Effector memory CD8+ T cells	0.3966 (0.1427–1.1024)	0.0762	142.5828 (4.0269–5048.491)	0.0064
MAIT cells	0.1485 (0.0492–0.4481)	7.00E-04	0.1747 (0.026–1.1741)	0.07
Chemokines activity	0.1640 (0.0708–0.3800)	2.46E-05	0.2526 (0.1051–0.6067)	0.002
Naive CD4 T cells	0.1838 (0.0625–0.5411)	0.0021		
Naive CD8 T cells	0.2160 (0.0692–0.6736)	0.0083		
CNV loss in TP53	0.8914 (0.8357–0.9509)	4.84E-04		
CNV loss in MAX	0.8170 (0.6846–0.9749)	0.0249		

## Discussion

T-cell infiltration into the TME is an important feature for the therapeutic activity and prognostic prediction (Spranger and Gajewski, 2018). However, the driver genes and signaling pathways regulating the T-cell infiltration have not been completely discovered in HCC. In the present study, we identified a series of cancer driver genes triggered by CNAs such as *CDKN2B*, *MYC*, *TSC1*, *TP53*, and *GSK3B*, which were enriched in the frequently amplified and deleted regions, respectively, and well-characterized in several cancers (Zack et al., 2013; Tokheim et al., 2016; Bailey et al., 2018). With the T-cell infiltration levels in liver normal tissues, HCC, and recurrent HCC tissues, we found T-cell infiltration levels were decreased progressively, showing consistency with previous studies that reduced T-cell infiltration might be associated with poor prognosis (Ye et al., 2019; Pinato et al., 2020).

Particularly, we observed that deletions of *MAX* and *TP53* were significantly associated with reduced T-cell infiltration in HCC. Loss of p53 function has been confirmed to decrease T-cell infiltration in breast cancer (Pinato et al., 2020), Accordingly, the downstream oncogenic pathway, cell cycle, was significantly activated in the low T-cell infiltration HCC, suggesting that the deletions in *MAX* or *TP53* regulated T-cell infiltration by enhancing uncontrolled cell cycle. As chemokines secreted by the tumor cells played vital roles in immune-cell recruitment in TME (Chow and Luster, 2014) and were transcriptionally regulated by transcription factors such as TP53 (Blagih et al., 2020), consistently, we found that these genes were highly enriched in the downregulated genes in HCC with TP53/MAX deletions, which gave us a hint that the CNV loss in TP53/MAX might downregulate their chemokine-relayed target genes, thereby weakening the immune-cell recruitment. As multiple biological processes were involved in T-cell–mediated anticancer activity, interestingly, we found that all the seven steps were reduced in low T-cell infiltration subgroup, indicating that cancer immunotherapy required drug combinations targeting these biological processes (Ott et al., 2017).

Furthermore, the T-cell infiltration levels and chemokines might be used as clinical biomarkers for the treatment response and prognostic prediction. Using the T-cell infiltration levels of five T cells and chemokine activity, we found that the T-cell infiltration levels and chemokine activity could accurately predict the response of sorafenib treatment. Similarly, immune cell abundance has been reported as an indicator for sorafenib response (Cabrera et al., 2013). In addition, the T-cell infiltration levels and chemokines were identified as favorable prognostic biomarkers in HCC, further suggesting that the T-cell infiltration levels were promising biomarkers to be applied in clinical practice.

In conclusion, we performed a systematic analysis to identify driver genes and signaling pathways involved in T-cell infiltration, which not only revealed the underlying mechanism that regulating T-cell infiltration, but also improved the understanding of the functional roles of T cells in HCC.

## Data Availability Statement

The datasets presented in this study can be found in online repositories. The names of the repository/repositories and accession number(s) can be found in the article/Supplementary Material.

## Author Contributions

YC, ZY, and JP contributed to the conception and design of the research. YC, YT, and JW contributed to the acquisition of data and analysis and interpretation of data. YC, YT, and JW contributed to statistical analysis. YC, YT, JW, WW, and QT contributed to drafting the manuscript. YC, YT, JW, LL, ZL, WL, YL, JP, and ZY contributed to revision of the manuscript. All authors contributed to the article and approved the submitted version.

## Conflict of Interest

The authors declare that the research was conducted in the absence of any commercial or financial relationships that could be construed as a potential conflict of interest.

## Supplementary Material

Supplementary Figure 1 The differential infiltrating levels of T cells across TNM stages and the differential expression levels of TP53 and MAX in Sorafenib responders and nonresponders.
