# Supplementary figures and images for: Identification of Driver Genes Regulating the T-Cell–Infiltrating Levels in Hepatocellular Carcinoma
Source: Front Genet. 2020 Dec 14;11:560546. doi: 10.3389/fgene.2020.560546 (PMC7767976; doi:10.3389/fgene.2020.560546)

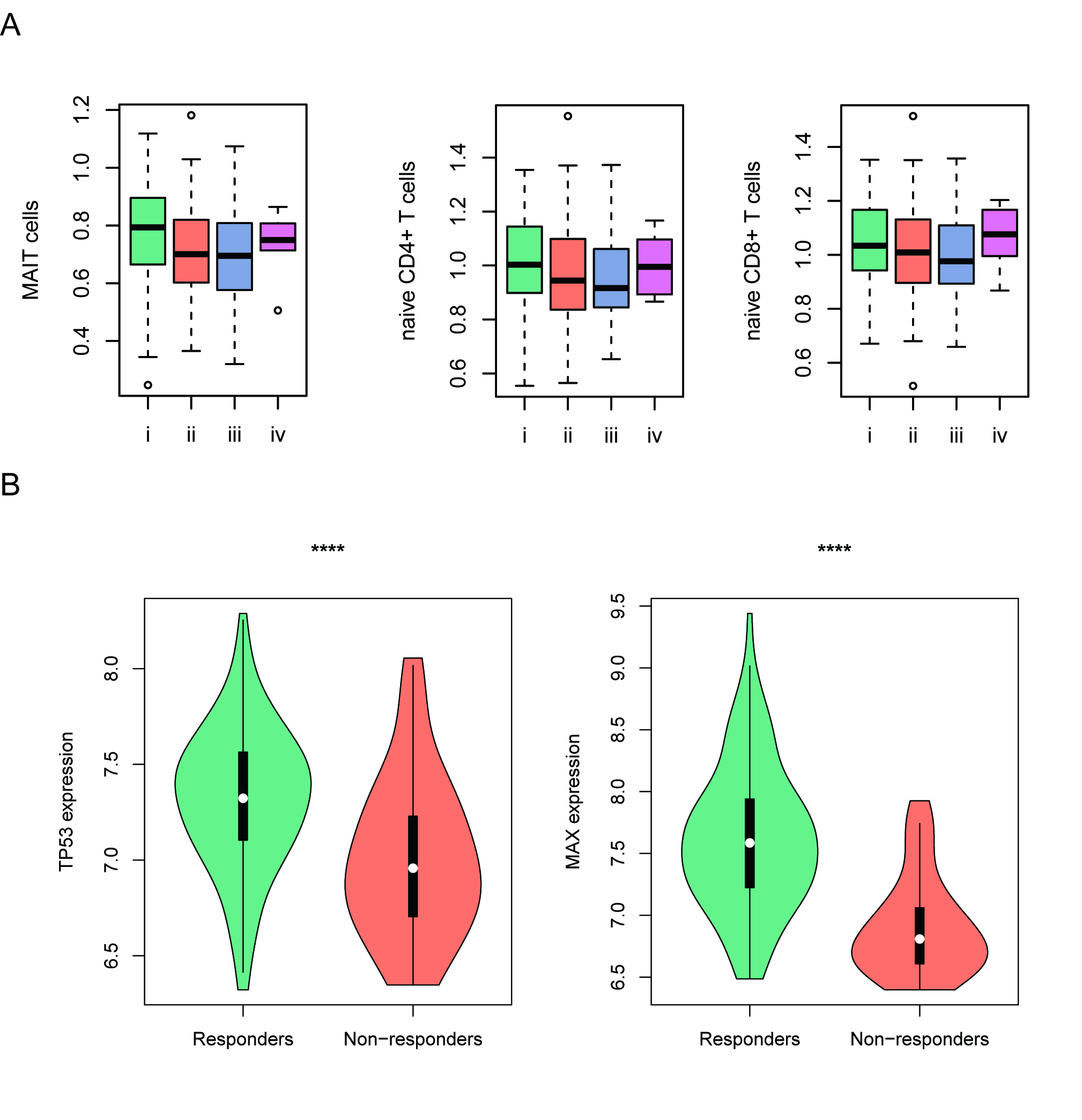

Supplement: Supplementary Figure 1 — The differential infiltrating levels of T cells across TNM stages and the differential expression levels of TP53 and MAX in Sorafenib responders and nonresponders. [file Image_1.TIF]
